# A Mutation in *Caenorhabditis elegans* NDUF-7 Activates the Mitochondrial Stress Response and Prolongs Lifespan via ROS and CED-4

**DOI:** 10.1534/g3.115.018598

**Published:** 2015-06-01

**Authors:** Manish Rauthan, Parmida Ranji, Ragda Abukar, Marc Pilon

**Affiliations:** Department of Chemistry and Molecular Biology, University of Gothenburg, Gothenburg, S-405 30, Sweden

**Keywords:** *C. elegans*, *ced-4*, *atfs-1*, aging, reactive oxygen species, statin, mevalonate pathway, UPR^mt^, mitochondria

## Abstract

The mevalonate pathway is responsible for the synthesis of cholesterol, coenzyme Q, and prenyl groups essential for small GTPase modification and function, and for the production of dolichols important for protein glycosylation. Statins, *i.e.*, cholesterol-lowering drugs that inhibit the rate-limiting enzyme in the mevalonate pathway, HMG-CoA reductase, are lethal to *Caenorhabditis elegans* even though this animal lacks the branch of the mevalonate pathway that leads to cholesterol synthesis. To better understand the effects of statins that are not related to cholesterol, we have adopted the strategy of isolating statin-resistant *C. elegans* mutants. Previously, we showed that such mutants often have gain-of-function mutations in ATFS-1, a protein that activates the mitochondrial unfolded protein response. Here, we describe the isolation of a statin-resistant mutant allele of the NDUF-7 protein, which is a component of complex I in the mitochondrial electron transport chain. The novel *nduf-7(et19)* mutant also exhibits constitutive and ATFS-1-dependent activation of the mitochondrial unfolded protein response (UPR^mt^) and prolonged life span, both of which are mediated through production of ROS. Additionally, lifespan extension, but not activation, of the mitochondrial unfolded protein response was dependent on the pro-apoptotic gene *ced-4*. We conclude that the *nduf-7(et19)* mutant allele causes an increase in reactive oxygen species that activate ATFS-1, hence UPR^mt^-mediated statin resistance, and extends life span via CED-4.

The mevalonate pathway is required for the synthesis of diverse biomolecules: cholesterol, an important membrane component as well as a precursor for several steroid hormones; coenzyme Q (CoQ), an antioxidant and part of the mitochondrial electron transport chain; isopentenyl adenosine, required for t-RNA modification; farnesyl diphosphate (FPP) and geranylgeranyl diphosphate (GGPP), lipid moieties needed for proper membrane association of proteins; and dolichols, essential for protein glycosylation (Goldstein and Brown 1990; Rauthan and Pilon 2011). The pathway has one main trunk and multiple sub-branches that synthesize the different metabolites (Figure 1A) (Rauthan and Pilon 2011). Inhibitors of this pathway, namely bisphosphonates and statins, are used in therapies to prevent loss of bone mass and to lower blood cholesterol levels (Buhaescu and Izzedine 2007) . Statins are the most widely used drugs to control cholesterol levels; they work by inhibiting 3-hydroxy-3-methylglutaryl coenzyme A reductase (HMG-CoA), a rate-limiting enzyme in the main trunk of the pathway. Statins have rare but adverse side effects ranging from severe muscle pain to massive muscle loss (rhabdomyolysis). The adverse effects of statins seem mostly unrelated to the lowered cholesterol levels and are more likely due to the limited production of other metabolites that depend on the mevalonate pathway for their synthesis (Harper and Jacobson 2007; Bełtowski *et al.* 2009).

**Figure 1:**
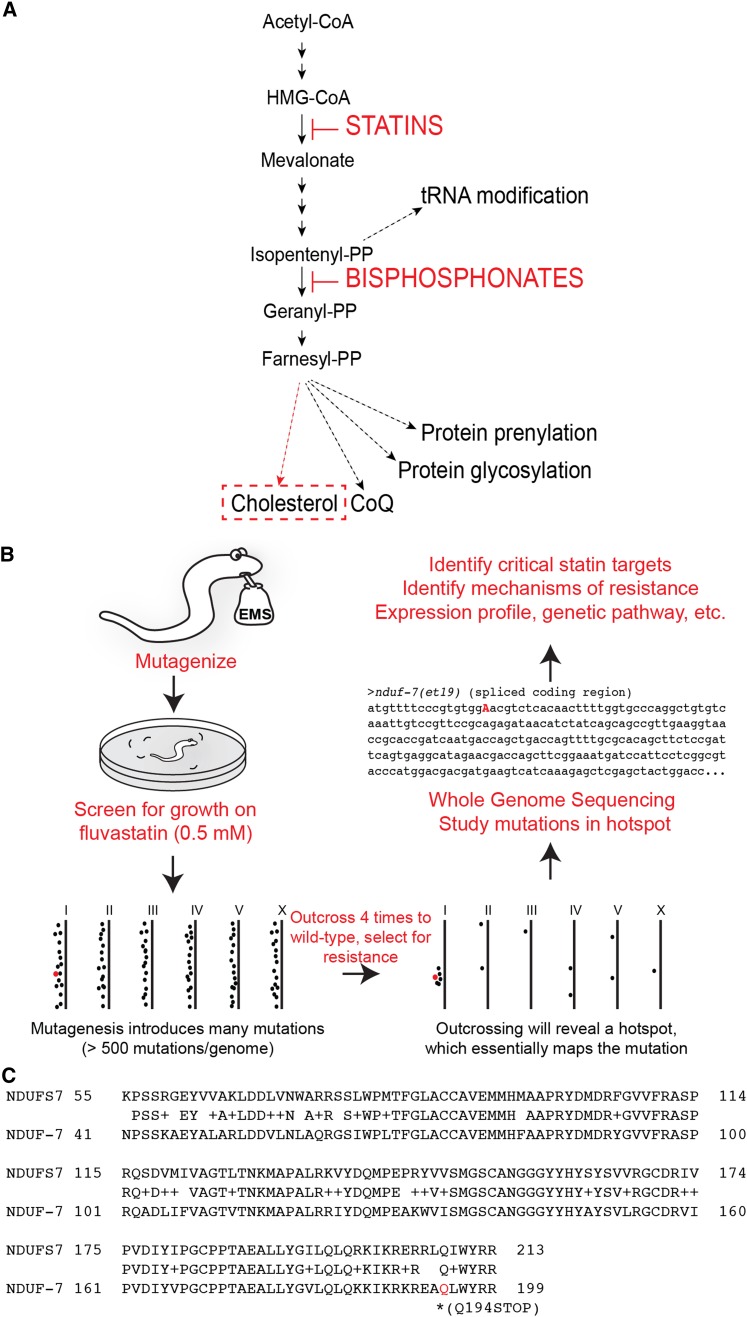
The mevalonate pathway and screening strategy leading to identification of the *nduf-7(et19)* allele. (A) Overview of the mevalonate pathway, its sub-branches, and site of action of two inhibitors, namely statins and bisphosphonates. (B) Outline of the screening strategy to isolate fluvastatin-resistant mutants and their identification through whole genome sequencing. (C) Alignment of the highest conserved region between NDUF-7 and its human homolog, NDUFS7. The mutation in the *nduf-7(et19)* mutant, *i.e.*, Q194STOP, is marked in red

*C. elegans* is an ideal model to study the noncholesterol effects of mevalonate pathway inhibition because this organism lacks the cholesterol synthesis branch but possesses all other branches (Figure 1A) (Rauthan and Pilon 2011). Previously, we have shown that inhibiting the mevalonate pathway in worms using statins results in larval lethality, and other phenotypes depending on the doses used, that can be fully rescued with exogenous mevalonate, thus demonstrating on-target effect of statins in worms (Mörck *et al.* 2009; Rauthan *et al.* 2013; Ranji *et al.* 2014). A forward genetic screen for statin resistance identified mitochondria as the primary site of its deleterious effects; mutants with gain-of-function (*gof*) alleles of ATFS-1, a key transcription factor required for activation of the mitochondrial stress response (Nargund *et al.* 2012), have a constitutively active mitochondrial unfolded protein response (UPR^mt^) and are resistant to statins (Rauthan *et al.* 2013). Importantly, inhibition of the mevalonate pathway prevents the activation of the UPR^mt^ in normal worms, which explains the necessity for UPR^mt^-activating mutations to achieve resistance (Ranji *et al.* 2014; Liu *et al.* 2014).

Here we show that a partial loss-of-function mutation in *nduf-7* (NADH-ubiquinone oxidoreductase Fe-S), which is a key component of the mitochondrial electron transport chain complex 1 (ETC-1), leads to constitutive activation of the UPR^mt^. *nduf-7(et19)* mutant worms have a reduced respiration rate and longer lifespan, and are resistant to two different types of statins. Furthermore, the constitutive UPR^mt^ activation in the *nduf-7(et19)* mutant requires ATFS-1 and is suppressed by reactive oxygen species scavengers, but not by mutations in *ced-4*, a pro-apoptotic gene required for the lifespan extension. We conclude that excessive ROS production due to impaired ETC-1 function in the *nduf-7(et19)* mutant causes activation of the UPR^mt^ and statin resistance, and extends lifespan via CED-4 .

## Materials and Methods

### Nematode strains and maintenance

All strains were maintained at 20° unless otherwise stated. The Bristol strain N2 was used as wild-type (WT) in all the experiments (Sulston and Hodgkin 1988). Strains with the following genotypes were obtained from the *Caenorhabditis* Genetics Center: *zcIs4[phsp4*::*GFP]*, *zcIs9[hsp-60*::*GFP]*, *ced-4(n1162)*, *isp-1(qm150)*, *dpy-5(e907)I*; *sEx[rCes W10D5.2*::*GFP + pCeh361]* (referred to as “*Pnduf-7*::*GFP*” in this article), and *atfs-1(gk3094)*. The strain *nduf-7(tm1436)* was provided by the MITANI Lab through the National Bio-Resource Project of the MEXT, Japan.

### Mutant screens

The mutagenesis screen to identify the statin-resistant mutant (*et19*) was performed as described previously (Rauthan *et al.* 2013). In short, N2 worms were mutagenized using ethyl methane sulfonate and L1 larvae from the F2 progeny were placed on 0.5 mM fluvastatin plates. Statin-resistant mutants were isolated by picking worms that could grow and reproduce within 4 to 5 d of placing them on the statin plates. These mutants were outcrossed six times with N2 worms and then sent for whole genome sequencing (WGS). The *et19* mutant was further outcrossed for a total of 10 times before performing any phenotypic study.

The *atfs-1(gof)* suppressor screen was performed by mutagenizing *atfs-1(et15) zcIs9[hsp60*::*GFP]* worms, where *hsp60*::*GFP* expression is constitutively active (Rauthan *et al.* 2013). Subsequently, GFP-negative worms were picked among the F2 progeny of the mutagenized animals. These suppressors were further scored for GFP expression and statin resistance.

### Whole genome sequencing

WGS was performed on *et19* mutant worms outcrossed six times as stated above. The identification of genetic hotspots and statin resistance-causing mutations in the *et19* worms was performed as described previously (Sarin *et al.* 2008; Zuryn *et al.* 2010; Rauthan *et al.* 2013).

### RNAi feeding experiments

RNAi knockdown of *nduf-7* and *atfs-1* was achieved by feeding worms with bacterial RNAi clones and seeded on IPTG plates according to a published protocol (Kamath 2003). Three to four L4 larvae were placed on these plates and allowed to grow and reproduce. Once their progeny reached adulthood, they were collected and bleached, and their eggs were allowed to hatch in M9 overnight. The resulting L1 larvae were then placed onto new RNAi plates. The length and GFP intensity of these worms were measured after 96 hr unless otherwise stated.

### Oxygen consumption assay

Oxygen consumption rates were measured using an Oxytherm (Hansatech) oxygen electrode as previously described (Schulz *et al.* 2007; Lee *et al.* 2009; Rauthan *et al.* 2013). A Pierce BCA Protein Assay Kit (Thermo Scientific) was used to measure protein concentration.

### Lifespan assay

All lifespan measurements were performed at 20°, as described previously (Rauthan *et al.* 2013; Ranji *et al.* 2014), starting with 1-d adults. In some experiments, N-Acetyl-L-cysteine (NAC) was added to the culture media prior to pouring the plates.

### Drug treatment

Plates with different concentrations of fluvastatin (brand Lescol; Novartis) were made according to the protocol described in previous studies (Mörck *et al.* 2009; Rauthan *et al.* 2013). Additional compounds used in this study were: rosuvastatin (Crestor; AstraZeneca); mevalonolactone (Sigma); ibandronate (Sigma); paraquat (Sigma); and NAC (Sigma). These were dissolved in water except for rosuvastatin, which was dissolved in DMSO.

### Length and GFP intensity measurement

Synchronized L1 larvae were placed on plates containing different concentrations of drugs or RNAi clones. After 48 or 96 hr, the worms were mounted on 2% agar pads containing 10 mM levamisole to paralyze them, and images were acquired using a Zeiss Axio Scope A1 to measure their length or to score GFP levels. All GFP images for a single experiment were taken with the same excitation intensity and exposure time. Length measurements and GFP intensity were determined using Image J (National Institutes of Health) (Schneider *et al.* 2012).

### Plasmids

#### Pnduf-7::nduf-7:

The genomic *nduf-7* gene along with its 3 kb of promoter and 1 kb of 3′ UTR was amplified from N2 genomic DNA using the following primer pairs: 5′- CTTGACCTCTGAAAATTGCGGGAAAC -3′ and 5′- GTGGGGCTTACTCGTACAAAATGAC -3′. The resulting PCR product was cloned in *pCR-Blunt II-TOPO XL* vector (Invitrogen).

### PCR scoring of the *nduf-7(tm1436)* allele

The *nduf-7(tm1436)* allele carries a 699-bp deletion that spans its second and third exon. The following primers were used to distinguish the WT from mutant loci, 5′- GCAGTCAGATTTTGAGTGCGT -3′ and 5′- CAAGCGATCGCCAGTAACAGC -3′, obtaining a 1371-bp band in WT and a 685-bp band for the mutant.

### Generation of transgenic worms

Germ line transformation was performed as described by Mello *et al.* (1991), and the dominant *rol-6(su1006)* allele was used as a marker for transgenic worms.

### Statistics

Unless stated otherwise, data points in graphs and columns in histograms show the average (n > 20), error bars show the SEM, and significant differences were determined using Student’s *t*-test.

## Results

### *et19* is a new statin-resistant mutant allele

**Figure 2:**
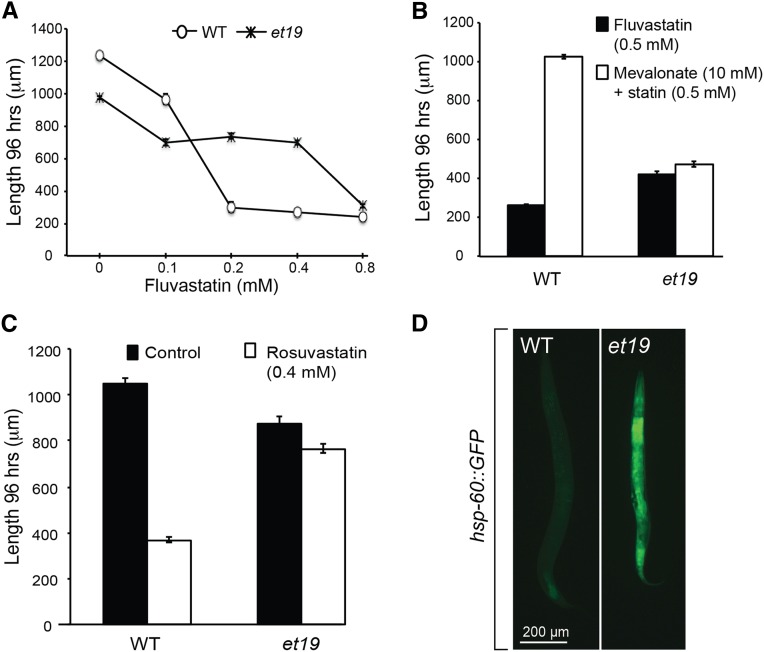
*nduf-7*(*et19)* mutant worms are resistant to statins and have activated mitochondrial UPR. (A) Length of wild-type and *nduf-7(et19)* mutant worms grown on varying concentrations of fluvastatin. (B) Effect of including 0.5 mM mevalonate on the length of wild-type or *nduf-7(et19)* mutants grown on 0.5 mM fluvastatin. (C) Length of wild-type and *et19* mutant worms grown on 0.4 mM rosuvastatin. (D) The *nduf-7(et19)* mutant constitutively expresses the UPR^mt^ reporter *hsp-60*::*GFP*.

The *et19* mutant allele was isolated in a forward genetic screen for mutants surviving on 0.5 mM fluvastatin plates **(**Figure 1B, Figure 2A**)**. At this concentration, WT worms cannot grow and arrest as L1 larvae. This deleterious effect of fluvastatin can be rescued by exogenously providing mevalonate, demonstrating that this is an on-target effect of the drug **(**Figure 2B**)**. Interestingly, the inclusion of mevalonate has no beneficial effects on the *et19* mutant worms grown on statin plates **(**Figure 2B**)**; this feature of the *et19* mutant is similar to previously isolated statin-resistant mutants that also did not benefit from the exogenous supply of mevalonate (Rauthan *et al.* 2013). The *et19* mutant worms are resistant to rosuvastatin (another class of statin), indicating that they have a generic resistance to statins rather than to one particular subtype **(**Figure 2C**)**. Previously, we showed that the UPR^mt^ is constitutively active in statin-resistant mutants and that preinduction of this response confers statin resistance in WT worms (Rauthan *et al.* 2013). We therefore hypothesized that the *et19* mutant may also be resistant to statins because of an activated mitochondrial stress response. Consistent with this hypothesis, *et19* mutant worms constitutively express high levels of *hsp-60*::*GFP*, a known marker of UPR^mt^ **(**Figure 2D**)** (Yoneda *et al.* 2004; Rauthan *et al.* 2013).

### Partial loss-of-function of *nduf-7* confers statin resistance

**Figure 3:**
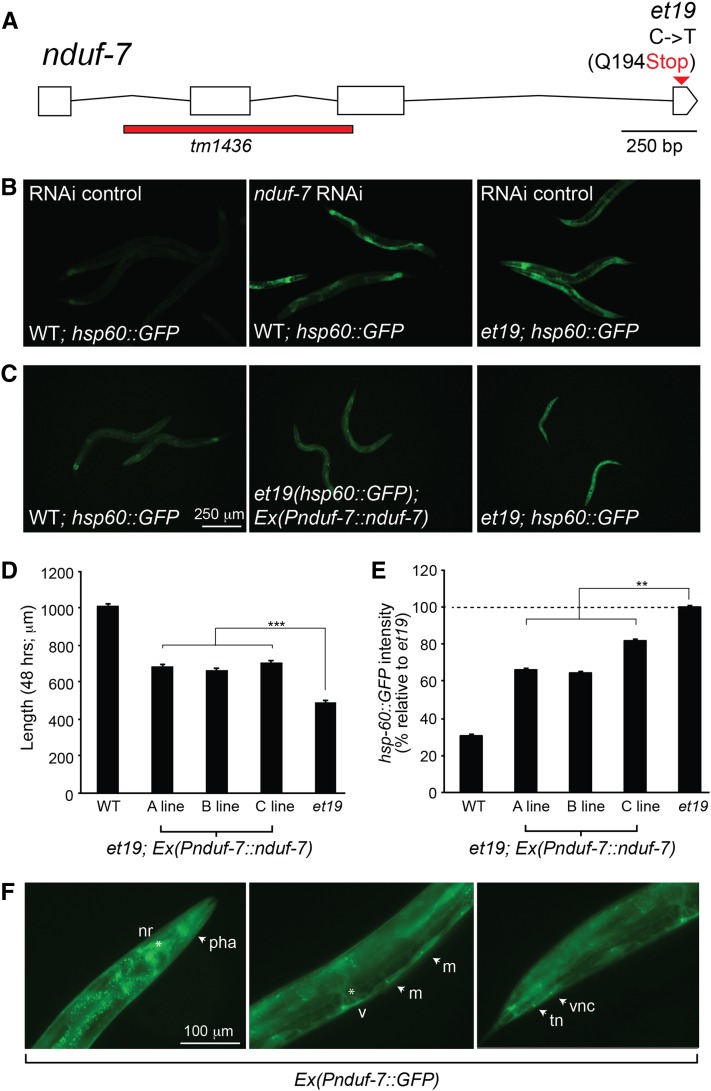
*nduf-7(et19)* is a partial loss-of-function allele of the *nduf-7* gene. (A) Structure of the *nduf-7* gene showing the position of the *et19* point mutation and of the *tm1436* deletion allele. (B) The *nduf-7(et19)* mutation or RNAi knockdown of the *nduf-7* gene in wild-type worms results in activation of the UPR^mt^ reporter *hsp-60*::*GFP*. (C) Transgenic *nduf-7(et19)* worms carrying a wild-type copy of the *nduf-7* gene (*Pnduf-7*::*nduf-7*) have decreased levels of *hsp-60*::*GFP* expression. (D and E) Quantification of the length and *hsp-60*::*GFP* expression in wild-type, *nduf-7(et19)*, and three *nduf-7(et19)* mutant lines carrying the wild-type *nduf-7* as a transgene (*Pnduf-7*::*nduf-7*). (F) Expression of the *Pnduf-7*::*GFP* transcriptional reporter. The following structures are indicated: pharynx (pha); body wall muscles (m); nerve ring (nr); vulva (v); ventral nerve cord (vnc); and tail neurons (tn). **P < 0.01 and ***P < 0.001 using Student’s *t*-test.

Using an outcrossing and WGS strategy (Sarin *et al.* 2008; Zuryn *et al.* 2010), we found that the *et19* mutant allele corresponds to a single nucleotide substitution mutation at the end of the *nduf-7* gene. This mutation introduces a premature STOP codon, resulting in a protein five amino acids smaller than the WT version **(**Figure 1C, Figure 3A**)**. NDUF-7 is a key subunit of the electron transport chain complex 1 and has 95.5% sequence homology to the human NDUFS7 protein (Tsang and Lemire 2003). The *nduf-7(et19)* is likely a partial loss-of-function allele because the more severe *nduf-7(tm1436)* allele, which lacks the second exon and part of the third exon (Figure 3A), is lethal (Supporting Information, Figure S1). Additionally, RNAi knockdown of the *nduf-7* gene in WT worms induces UPR^mt^ activation (Figure 3B). Furthermore, knocking down *nduf-7* in the *et19* mutant worms results in larval arrest, suggesting that NDUF-7 is partially functional in this mutant and that RNAi lowers its activity below an essential threshold. Additional proof that *et19* is a mutant allele of *nduf-7* is that expressing a WT copy of this gene in the *et19* mutant suppresses *hsp-60*::*GFP* expression and rescues the growth defect **(**Figure 3, C–E**)**, and that the same transgene can rescue the lethality of the *nduf-7(tm1436)* deletion mutant (Figure S1). To examine the expression pattern of *nduf-7*, we studied transgenic worms carrying the transcriptional reporter *Pnduf-7*::*GFP*. As could be expected from the mitochondrial function of the gene, the *nduf-7* reporter is ubiquitously expressed, with strongest expression in the pharynx, the nerve ring, the body wall muscle, and the intestine in comparison to other tissues **(**Figure 3F**)**.

### The UPR^mt^ is activated via ATFS-1 in the *nduf-7(et19)* mutant

**Figure 4:**
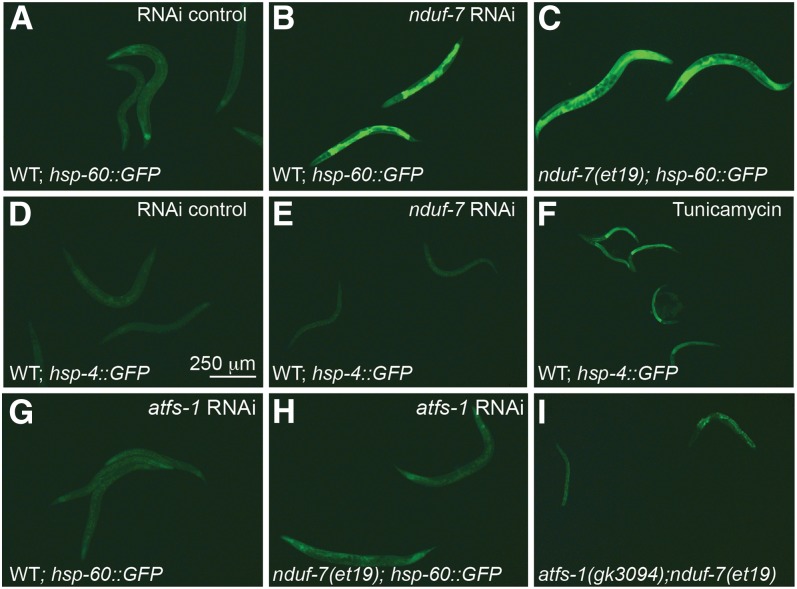
Activation of the UPR^mt^ in the *nduf-7(et19)* mutant is mediated through ATFS-1. (A–F) RNAi knockdown of *nduf-7* as well as the *nduf-7(et19)* mutation activate the UPR^mt^ reporter *hsp-60*::*GFP*, but not the UPR^er^ reporter *hsp-4*::*GFP*; tunicamycin is used as a positive control of *hsp-4*::*GFP* activation in (F). (G and H) UPR^mt^ activation in the *nduf-7(et19)* mutants is suppressed by *atfs-1* knockdown. (I) *atfs-1* is required for the viability of the *nduf-7(et19)* because the double mutant worms have severe growth defects and arrest as young larvae (two are shown).

Mutations in ETC subunits often cause activation of the mitochondrial stress machinery (Durieux *et al.* 2011; Nargund *et al.* 2012; Khan *et al.* 2013). Likewise, both *the nduf-7(et19)* mutation and RNAi against *nduf-7* cause activation of the UPR^mt^ reporter *hsp-60*::*GFP* (Figure 4, A and B). The stress response is specific for mitochondria because neither the knockdown of *nduf-7* nor the *nduf-7(et19)* allele causes activation *hsp-4*::*GFP*, a reporter of the unfolded protein response in the endoplasmic reticulum (UPR^er^) (Kapulkin *et al.* 2005). Furthermore, activation of the UPR^mt^ in the *nduf-7(et19)* mutants does not occur when the *atfs-1* gene is inhibited by RNAi knockdown (Figure 4, G and H). This suggests that the UPR^mt^ activation in the *nduf-7(et19)* mutant occurs via ATFS-1. UPR^mt^ activation is essential for the viability of the *nduf-7(et19)* mutant, because the double mutant *atfs-1(gk3094);nduf-7(et19)* is very sick and arrests as early larvae (Figure 4I).

### ATFS-1 likely regulates directly the *hsp-60*::*GFP* reporter

**Figure 5:**
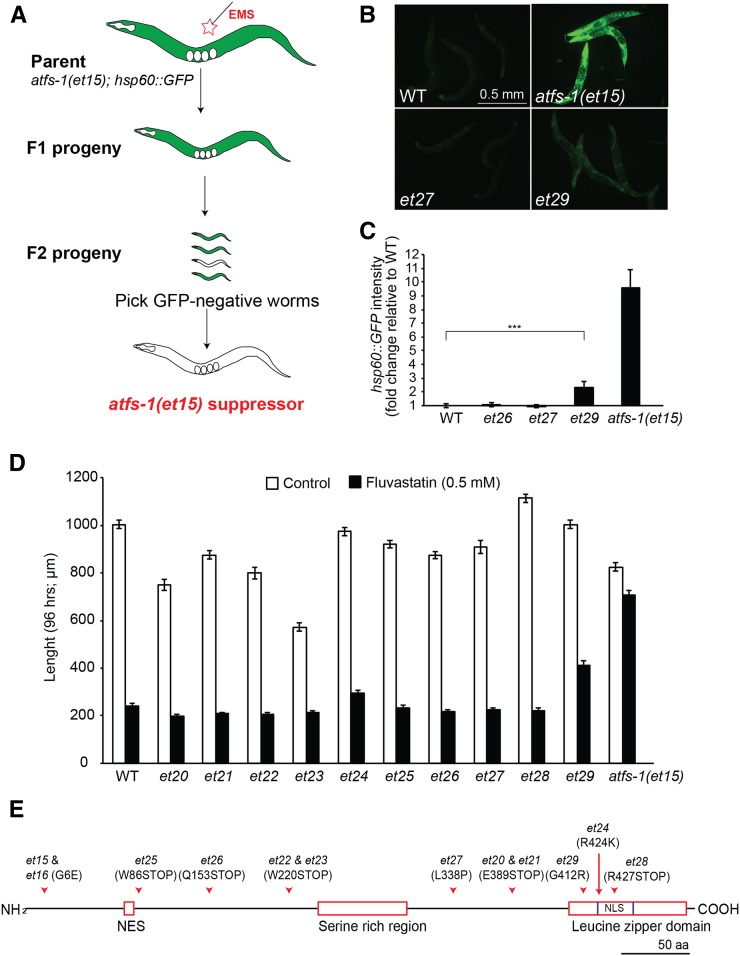
Characterization of *atfs-1(et15)* suppressors. (A) Overview of the screening strategy: *atfs-1(et15);zcIs9(Phsp60*::*GFP)* worms were mutagenized for 4 hr by incubation in the presence of 0.5% ethyl methane sulfonate, and approximately 27,000 mutagenized haploid genomes were screened among the F2 progeny, leading to the isolation of 14 GFP-negative mutants. (B) Images of *hsp-60*::*GFP* expression in control worms, *atfs-1(et15)* worms, and the *atfs-1(et15)* suppressors *et27* and *et29*. (C) Quantification of the fluorescence levels for representative *atfs-1(et15)* suppressor mutants. Note that the *et29* allele retains some *hsp-60*::*GFP* expression. (D) Length of 10 *atfs-1(et15)* suppressors 96 hr after being deposited as L1s on culture plates containing 0.5 mM fluvastatin; the *atfs-1(et15)* is also included as a control. Note that the *et29* allele retains a partial resistance to fluvastatin. (E) Molecular definition of the *atfs-1(et15)* suppressors. The main structural features of the ATFS-1 protein are depicted, and the nature and position of the alleles used in this study are indicated. For each allele, the *atfs-1* coding regions were PCR-amplified and both strands were sequenced to define the mutation.

The genetic pathway linking *nduf-7(et19)* and the UPR^mt^ reporter *hsp-60*::*GFP* likely involves no additional components between *atfs-1* and its target *hsp-60* promoter; a forward genetic screen to identify suppressors of the previously identified *atfs-1(gof)* allele *et15*(Rauthan *et al.* 2013) resulted in the isolation of 14 intragenic loss-of-function alleles of *atfs-1* itself (Figure 5, A–C). Ten of these mutants were characterized in some detail. All were growth-inhibited by 0.5 mM fluvastatin, to which the *atfs-1(et15)* allele is resistant, and all harbored mutations within the coding region of *atfs-1* (Figure 5, D and E). There are at least three possible explanations for having isolated only *atfs-1* intragenic suppressors in this screen: (1) *atfs-1* may act directly on the *hsp-60* promoter; (2) *atfs-1* may act together with essential genes that cause lethality when mutated; and (3) *atfs-1* may act together with any of several redundant genes. In any case, it may be difficult to further investigate the pathway between *atfs-1* and its target promoter using a forward genetics approach. We therefore focused our effort on better understanding how the *nduf-7(et19)* mutation leads to *atfs-1* activation.

### The extended lifespan of the *nduf-7(et19)* mutant is mediated through ROS signaling and CED-4

**Figure 6:**
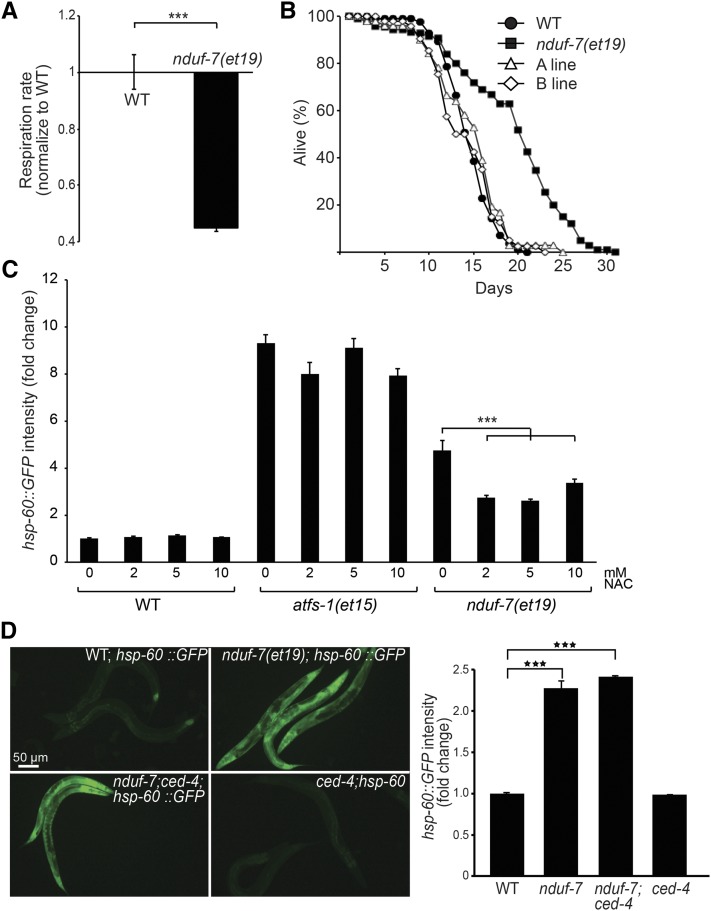
The *nduf-7(et19)* mutant has defective respiration, extended lifespan, and requires ROS but not *ced-4* for UPR^mt^ activation. (A) *nduf-7(et19)* mutant worms have reduced respiration rate. (B) *nduf-7(et19)* mutant worms have an extended lifespan that is suppresses when these worms carry wild-type *nduf-7* as a transgene (A and B lines). (C) Constitutive *hsp-60*::*GFP* expression is suppressed by nicotininc acid (NAC) in *nduf-7(et19)* mutant but not in *atfs-1(et15)* mutant worms. ***P < 0.001 using Student’s *t*-test. (D) The *ced-4(n1162)* mutation has no effect on its own or on the constitutive expression of *hsp-60*::*GFP* in the *nduf-7(et19)* mutant.

**Figure 7:**
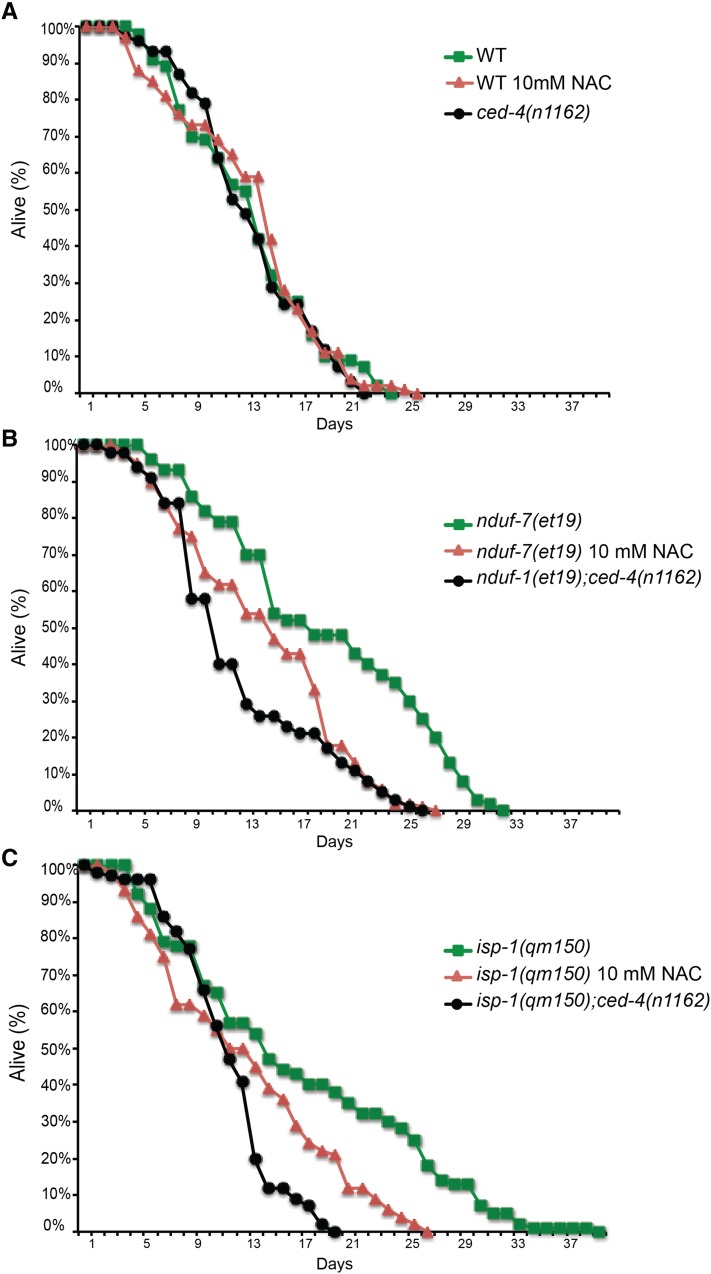
The extended lifespans of the *nduf-7(et19)* and *isp-1(qm150)* mutants are suppressed by the antioxidant NAC and by a *ced-4* mutation. NAC treatment or inclusion of the *ced-4* mutation had no effect on wild-type worms (A) but caused a reduction in lifespan in the *isp-1(qm150)* and *nduf-7(et19)* mutants (*P* < 0.001 except for *isp-1;ced-4*, which differed from the *isp-1* single mutant with a significance of *P* < 0.005). (B and C). The *isp-1* mutant was included as a control for the efficacy of the NAC treatment and for the effect of the *ced-4(n1162)* mutation.

Partial loss-of-function mutations in genes that encode components of the ETC, such as *nuo-6* and *isp-1*, often result in lifespan extension (Yang and Hekimi 2010a; Khan *et al.* 2013). This is mediated through decreased electron transport and excess production of reactive oxygen species (ROS) by mitochondria (Yang and Hekimi 2010a; Hwang and Lee 2011). Similarly, the *nduf-7(et19)* mutant has a low respiration rate, indicative of a compromised ETC function, as well as an extended lifespan **(**Figure 6, A and B**)**. Elevated ROS levels appear essential for both the UPR^mt^ activation and lifespan elongation in the *nduf-7(et19)* mutant because these phenotypes are abrogated by NAC, a hydrophilic antioxidant against all types of ROS (Aruoma *et al.* 1989; Benrahmoune *et al.* 2000; Yang and Hekimi 2010a) (Figure 6C, Figure 7). Importantly, NAC had no effect on the UPR^mt^ activation of the *atfs-1(et15) gof* mutant (Figure 6C). Our results suggest that UPR^mt^ activation and lifespan elongation in the *nduf-7(et19)* mutant are dependent on elevated ROS, which may act upstream of ATFS-1.

Is UPR^mt^ activation sufficient to explain the lifespan extension of the *nduf-7(et19)* mutant, as has been suggested for other long-lived mutants (Durieux *et al.* 2011; Houtkooper *et al.* 2013)? If that were the case, then it should be difficult to genetically separate the UPR^mt^ activation from the lifespan extension. To explore this question, we tested the effect of a *ced-4* mutation on these two phenotypes of the *nduf-7(et19)* mutant. CED-4, best known for its role as an activator of the cell-killing caspase CED-3 in programmed cell death, has recently been implicated in lifespan extension by ECT mutants, such as *isp-1(qm150)* (Yee *et al.* 2014). We found that *nduf-7(et19);ced-4(n1162)* double mutants retain the activated UPR^mt^ and statin-resistance phenotypes of the *nduf-7(et19)* single mutant (Figure 6D and Figure S2**)**, but do not show its extended lifespan phenotype (Figure 7). We conclude that UPR^mt^ activation is not sufficient to account for the lifespan extension in the *nduf-7(et19)* mutant.

## Discussion

The present study provides yet another line of support for the idea that the most effective way to overcome mevalonate pathway inhibition is to activate the UPR^mt^. We now know of three ways by which UPR^mt^ can be activated to confer resistance to mevalonate pathway inhibition. First, preinducing the UPR^mt^ through drugs such as paraquat or ethidium bromide confers resistance against mevalonate pathway inhibition (Rauthan *et al.* 2013). Second, gain-of-function mutation in ATFS-1 results in constitutive activation of the UPR^mt^ and gives resistance against mevalonate pathway inhibitors (Rauthan *et al.* 2013). Third, here we have elucidated a mechanism where partial loss-of-function in the *nduf-7* gene, a subunit of the ETC-1, causes constitutive activation of the UPR^mt^. How the UPR^mt^ circumvents mevalonate pathway inhibition is far from clear. It is possible that the UPR^mt^ allows an essential degree of mitochondrial function and homeostasis to be maintained even when active small GTPases, which are otherwise important for organelle homeostasis, are in limited supply when the output of prenyl groups from the mevalonate pathway is drastically reduced.

Paradoxically, genetic or pharmacological inhibition of the mevalonate pathway also prevents UPR^mt^ activation (Ranji *et al.* 2014; Liu *et al.* 2014). The mechanism behind this phenomenon is not clear, although impairment of small GTPases dependent on the mevalonate pathway for their prenylation have been implicated (Rauthan *et al.* 2013). In any case, gain-of-function mutations in ATFS-1 or, as shown here, mutations in *nduf-7* that act in an ATFS-1-dependent way do cause UPR^mt^ activation even in statin-treated worm. This suggests that the mevalonate pathway is usually required for events upstream of ATFS-1 during UPR^mt^ activation.

The mutation in *nduf-7(et19)* results in a mutant protein lacking the five C-terminal amino acids. These amino acids are highly conserved between the worm protein and its human homolog, *NDUFS7*, suggesting functional importance (Tsang and Lemire 2003). Multiple point mutations in *NDUFS7* are associated with Leigh syndrome, a heterogeneous neurological genetic disorder caused by mutations in ETC components and characterized by tell-tale brain morphology defects as well as abnormal findings in the mitochondria of skeletal muscles (Finsterer 2008). NDUFS7 is one of the subunits of the ubiquinone reduction module (Q module), which is the main catalytic unit of the mitochondrial ETC-1 (Triepels *et al.* 1999; Lebon *et al.* 2007). Mutations in NDUFS7 are presumed to affect either the catalytic activity or proper assembly of complex-1. In particular, the C-terminus of the NDUFS7 protein is important for proper interaction with its adjoining subunits and is critical for its assembly (Mimaki *et al.* 2012). Loss of the last five amino acids, as in the *nduf-7(et19)* allele, likely results in improper assembly and reduced function of ETC-1, which then leads to mitochondrial stress. This correlates well with our results, which show that the *nduf-7(et19)* mutant worms grow slower, have reduced respiration, and have a constitutively activated UPR^mt^.

The novel *nduf-7(et19)* allele described here joins a group of mutations that impair mitochondrial function and expand lifespan in *C. elegans*. Included in this group are alleles of the coenzyme Q biosynthetic protein CLK-1 (Lakowski and Hekimi 1996; Felkai *et al.* 1999), of the NUO-6 protein that is homologous to the vertebrate NDUFB4/B15 in complex I (Yang and Hekimi 2010b), of the Rieske iron sulfur protein ISP-1 in complex III (Feng *et al.* 2001), and of the thiamine pyrophosphokinase TPK-1 (de Jong *et al.* 2004; Butler *et al.* 2013; reviewed in Dancy *et al.* 2014). *C. elegans* mutants that harbor mutations in ETC subunits have increased ROS levels that contribute to their longevity (Yang and Hekimi 2010a). ROS, including superoxides, can act as intracellular messengers impinging on different signaling pathways to regulate biological processes such as cell proliferation and differentiation or inflammatory responses (Holmström and Finkel 2014). Elevated ROS production can also inflict oxidative damage on cell components, and maintaining ROS levels within a physiologically acceptable range is essential for viability. This is done either by inhibiting the cellular sources of ROS or through the expression of proteins that detoxify superoxides, namely superoxide dismutases (Yang and Hekimi 2010a; Holmström and Finkel 2014). Our observation that the antioxidant NAC prevents UPR^mt^ activation in the *nduf-7(et19)* mutant suggests that elevated ROS not only are the outcome of impaired ETC function but also act as a signal to activate the UPR^mt^ in the mutant. This is an important observation because an alternative mechanism could have been that a mutated NDUF-7 protein results in protein misfolding or aggregates that trigger UPR^mt^ independently of ROS levels. That *nduf-7(et19)* triggers UPR^mt^ via the elevated ROS levels suggests the existence of a feedback loop to control the level of ROS in mitochondria by upregulating the expression of detoxifying genes that are part of the UPR^mt^ response. Additionally, increased ROS production promotes cellular changes that attenuate the effects of aging. Long-lived mutants such as *isp-1* and *nuo-6*, which harbor mutations in ETC subunits, have elevated superoxide levels that contribute to their extended lifespan phenotypes (Yang and Hekimi 2010a; Dancy *et al.* 2014). Similarly, the extended lifespan of the *nduf-7(et19)* mutant depends on elevated ROS because it is suppressed by the inclusion of an antioxidant in the culture plates. Interestingly, the effect of ROS on UPR^mt^ and longevity can be separated genetically. Specifically, we found that *ced-4*, a gene that is part of the apoptotic pathway and recently found to have a separate role in contributing to the longevity of ETC mutants (Yee *et al.* 2014), is also required for the longevity of the *nduf-7(et19)* mutant but is not required for the constitutive activation of the UPR^mt^ and statin resistance. In other words, the UPR^mt^ is not sufficient for lifespan extension in the *nduf-7(et19)* mutant, a conclusion in agreement with other studies that directly addressed the role of the UPR^mt^ in lifespan extension (Bennett *et al.* 2014; reviewed in Bennett and Kaeberlein 2014). We previously showed that ATFS-1 mutations that cause constitutive UPR^mt^ actually reduce lifespan in *C. elegans* (Rauthan *et al.* 2013), suggesting that although a moderate activation of the UPR^mt^ can be protective and beneficial in some contexts, sustained/elevated UPR^mt^ is likely deleterious.

## Supplementary Material

Supporting Information
