# Supplementary material for: A Mutation in Caenorhabditis elegans NDUF-7 Activates the Mitochondrial Stress Response and Prolongs Lifespan via ROS and CED-4
Source: G3 (Bethesda). 2015 Jun 1;5(8):1639–48. doi: 10.1534/g3.115.018598 (PMC4528320; doi:10.1534/g3.115.018598)
Supplement: Supporting Information [file supp_5_8_1639__index.html]

A Mutation in Caenorhabditis elegans NDUF-7 Activates the Mitochondrial Stress Response and Prolongs Lifespan via ROS and CED-4 — Supporting Information 

# A Mutation in *Caenorhabditis elegans* NDUF-7 Activates the Mitochondrial Stress Response and Prolongs Lifespan via ROS and CED-4

## Supporting Information for Rauthan *et al.*, 2015

**Files in this Data Supplement:**

- Supporting Information - Figures S1-S2 (PDF, 965 KB)
- Figure S1 - The *nduf-7(tm1436)* deletion mutant is lethal. (PDF, 826 KB)
- Figure S2 - The *nduf-7(et19)* mutation activates the UPRmt independently from *ced-4*. (PDF, 246 KB)
