# Supplementary material for: A Mutation in Caenorhabditis elegans NDUF-7 Activates the Mitochondrial Stress Response and Prolongs Lifespan via ROS and CED-4
Source: G3 (Bethesda). 2015 Jun 1;5(8):1639–48. doi: 10.1534/g3.115.018598 (PMC4528320; doi:10.1534/g3.115.018598)
Supplement: Supporting Information [file supp_g3.115.018598_018598SI.pdf]

G3 · Genes | Genomes | Genetics

A Mutation in *C. elegans* NDUF-7 Activates the Mitochondrial Stress Response, and Prolongs Lifespan via ROS and CED-4

Manish Rauthan, Parmida Ranji, Ragda Abukar and Marc Pilon

Author affiliation:

Department of Chemistry and Molecular Biology,

University of Gothenburg, Gothenburg, S-405 30, Sweden.

Corresponding author:

Marc Pilon, Department of Chemistry and Molecular Biology,

University of Gothenburg, Medicinaregaran 9C, S-405 30 Sweden.

Email: marc.pilon@cmb.gu.se.

TEL: +46 31 786 3279.

FAX: +46 31 786 3801.

DOI: 10.1534/g3.115.018598

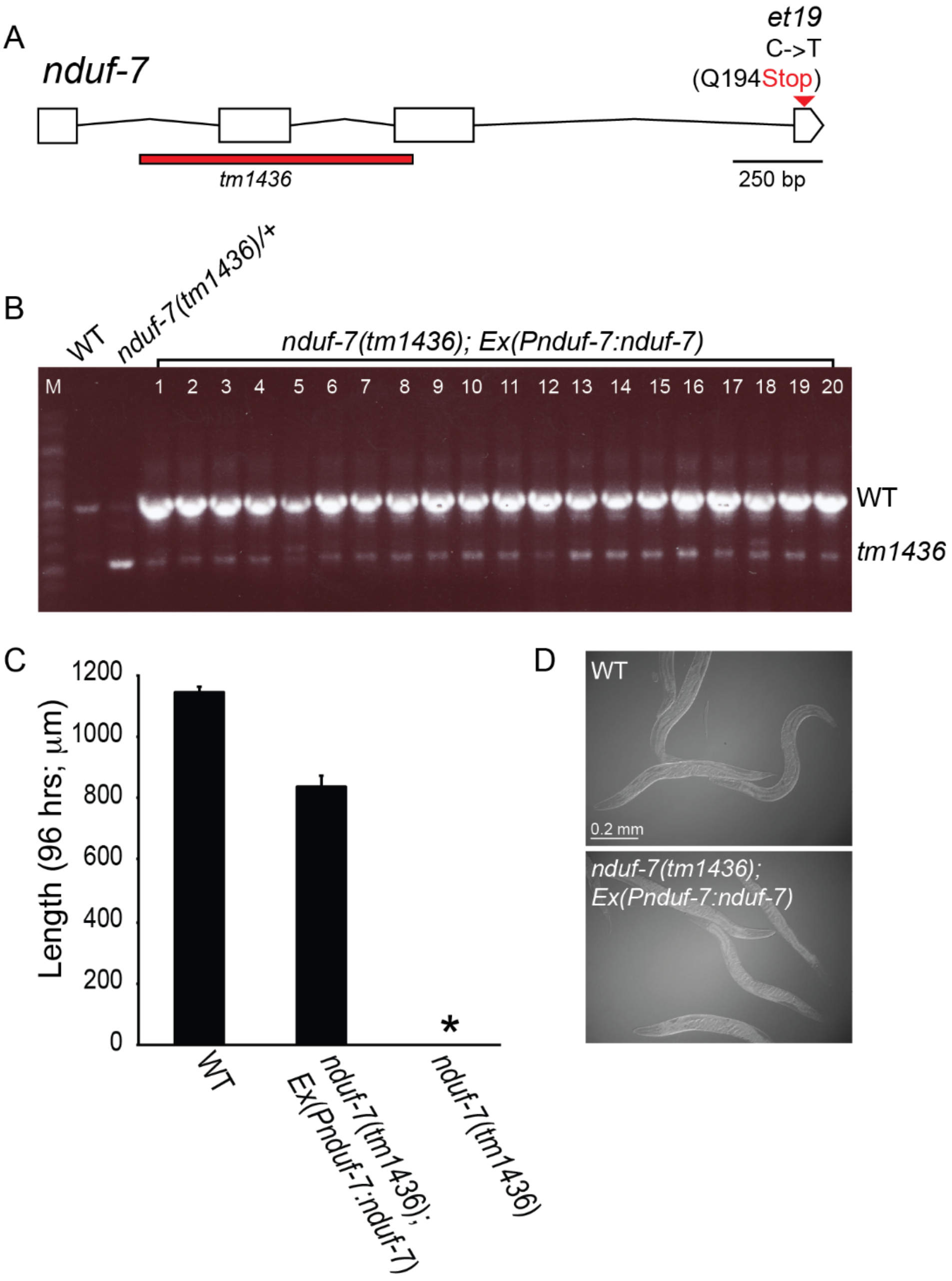

Figure S1 The *nduf-7(tm1436)* deletion mutant is lethal. (A) Structure of the *nduf-7* gene with the positions of the *tm1436* deletion and *et19* point mutation indicated. (B) DNA from 20 randomly picked progeny of homozygous *nduf-7(tm1436)* mutant worms carrying the wild-type *nduf-7* gene on a extrachromosomal array was amplified using primers flanking the *tm1436* deletion: all carried the transgene even though several progeny are normally expected to lack the extrachromosomal array Indeed, no *tm1436* homozygous worm was ever found that did not also carry the rescuing transgene, indicating that it is required for their viability. Wild-type and *tm1436* heterozygous worms were used as controls in the two lanes next to the molecular weight marker lane, M. (C-D) Length measurement and images of the homozygous *nduf-7(tm1436)* mutant worms rescued by the wild-type *nduf-7* transgene. The asterisk in (C) indicates no viability, hence no growth, in non-transgenic mutant worms.

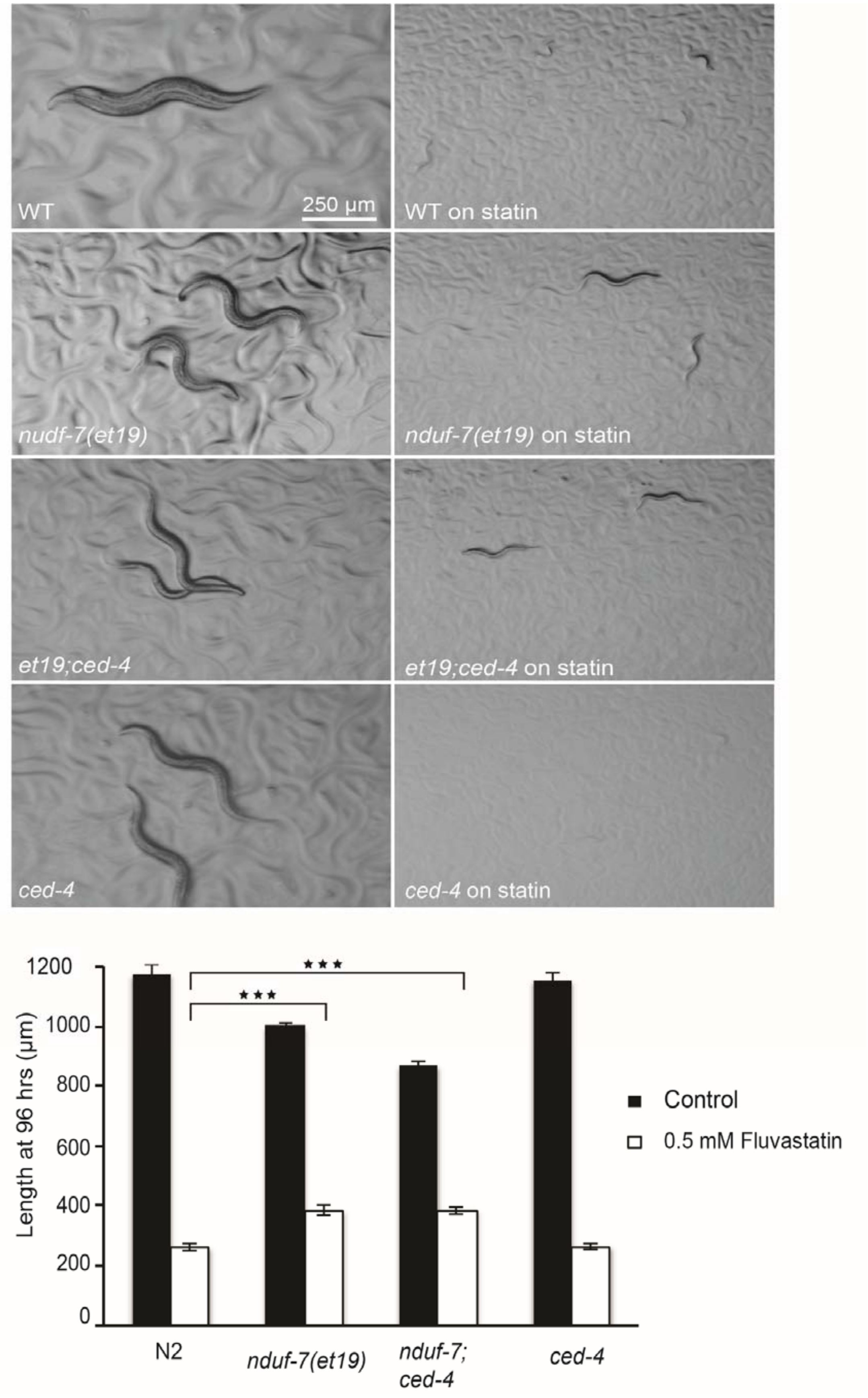
WT
250 μm
WT on statin
nudf-7(et19)
nduf-7(et19) on statin
et19;ced-4
et19;ced-4 on statin
ced-4
ced-4 on statin
Length at 96 hrs (μm)
1200
1000
800
600
400
200
0
★★★
★★★
Control
0.5 mM Fluvastatin
N2
nduf-7(et19)
nduf-7;
ced-4
ced-4

Figure S2 The *nduf-7(et19)* mutation activates the UPR^{mt} independently from *ced-4.* (A) Images of worms with the indicated genotypes dispensed as L1s onto culture plates with or without 0.5 mM fluvastatin then cultivated for 96 hours. (B) The graphs shows the average lengths from at least 20 worms for each condition. Note that the presence of the *ced-4* mutation did not reduce the statin resistance in the *nduf-7(et19)* mutant. *** indicates p< 0.001 in a Student's t-test.
