# Supplementary material for: A Mutation in Caenorhabditis elegans NDUF-7 Activates the Mitochondrial Stress Response and Prolongs Lifespan via ROS and CED-4
Source: G3 (Bethesda). 2015 Jun 1;5(8):1639–48. doi: 10.1534/g3.115.018598 (PMC4528320; doi:10.1534/g3.115.018598)
Supplement: Supporting Information [file supp_g3.115.018598_FigureS1.pdf]

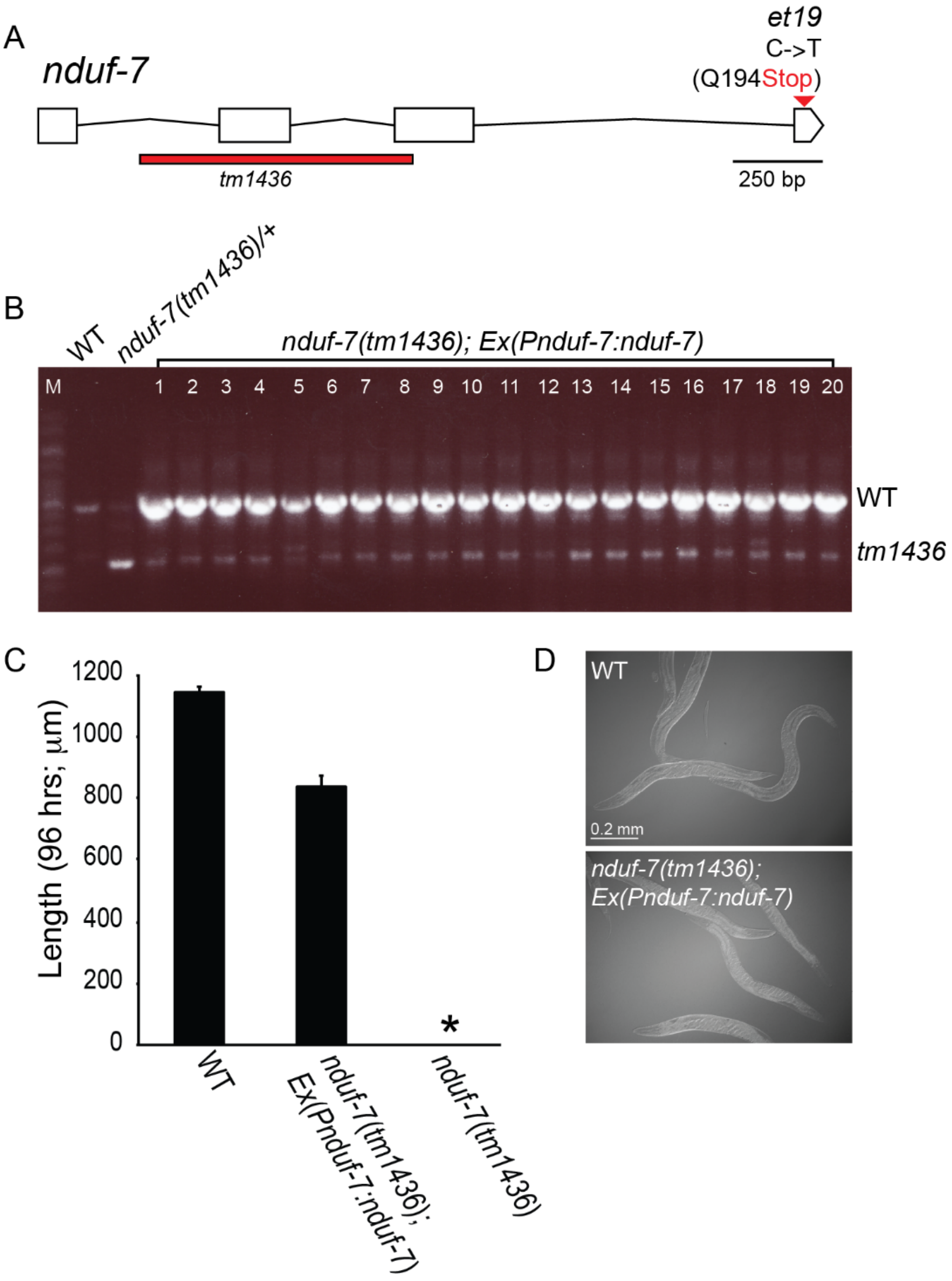

Figure S1 The *nduf-7(tm1436)* deletion mutant is lethal. (A) Structure of the *nduf-7* gene with the positions of the *tm1436* deletion and *et19* point mutation indicated. (B) DNA from 20 randomly picked progeny of homozygous *nduf-7(tm1436)* mutant worms carrying the wild-type *nduf-7* gene on a extrachromosomal array was amplified using primers flanking the *tm1436* deletion: all carried the transgene even though several progeny are normally expected to lack the extrachromosomal array Indeed, no *tm1436* homozygous worm was ever found that did not also carry the rescuing transgene, indicating that it is required for their viability. Wild-type and *tm1436* heterozygous worms were used as controls in the two lanes next to the molecular weight marker lane, M. (C-D) Length measurement and images of the homozygous *nduf-7(tm1436)* mutant worms rescued by the wild-type *nduf-7* transgene. The asterisk in (C) indicates no viability, hence no growth, in non-transgenic mutant worms.
