# Supplementary material for: A Mutation in Caenorhabditis elegans NDUF-7 Activates the Mitochondrial Stress Response and Prolongs Lifespan via ROS and CED-4
Source: G3 (Bethesda). 2015 Jun 1;5(8):1639–48. doi: 10.1534/g3.115.018598 (PMC4528320; doi:10.1534/g3.115.018598)
Supplement: Supporting Information [file supp_g3.115.018598_FigureS2.pdf]

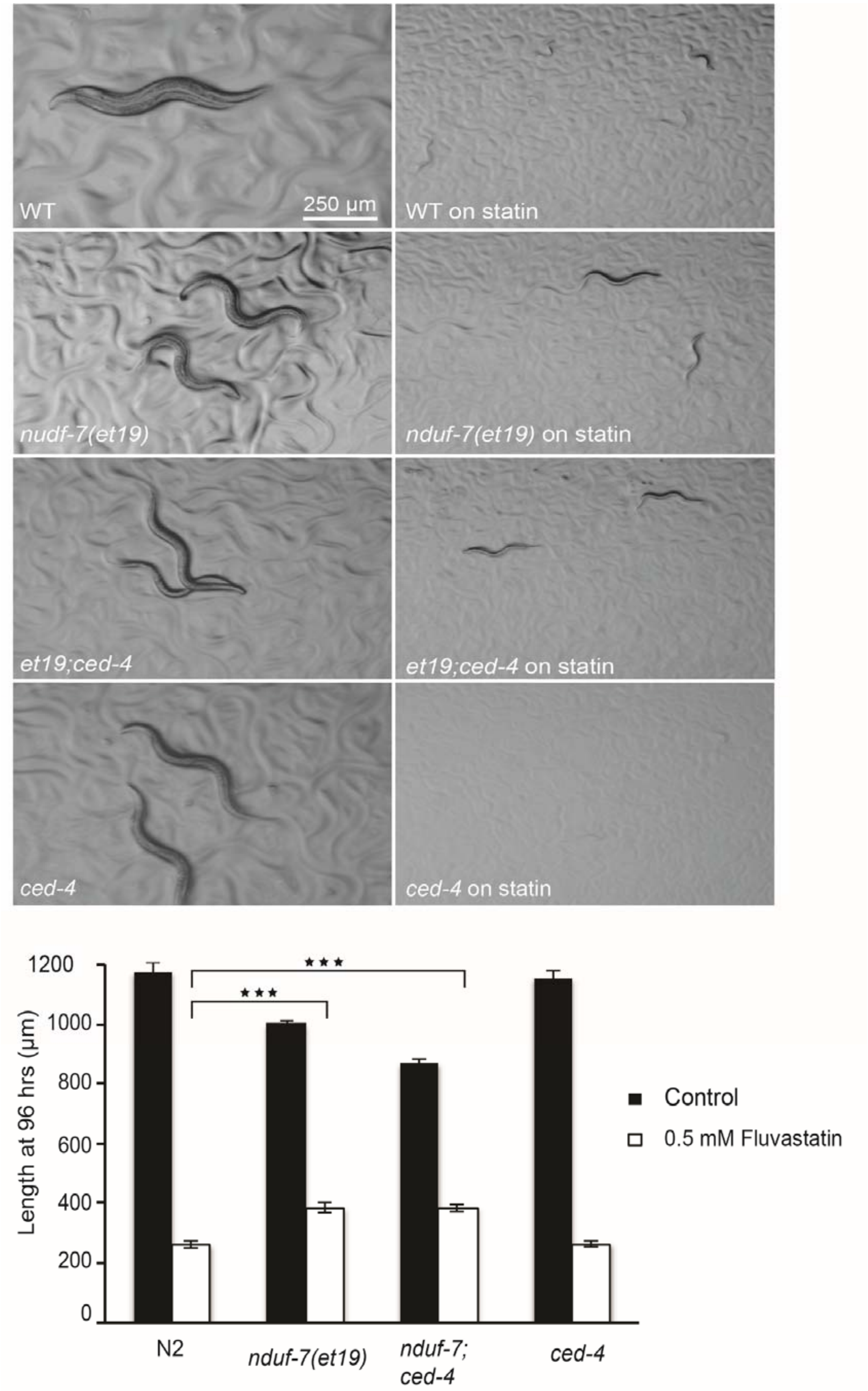
WT
250 μm
WT on statin
nudf-7(et19)
nduf-7(et19) on statin
et19;ced-4
et19;ced-4 on statin
ced-4
ced-4 on statin
Length at 96 hrs (μm)
1200
1000
800
600
400
200
0
***
***
Control
0.5 mM Fluvastatin
N2
nduf-7(et19)
nduf-7;
ced-4
ced-4

Figure S2 The *nduf-7(et19)* mutation activates the UPR^{mt} independently from *ced-4.* (A) Images of worms with the indicated genotypes dispensed as L1s onto culture plates with or without 0.5 mM fluvastatin then cultivated for 96 hours. (B) The graphs shows the average lengths from at least 20 worms for each condition. Note that the presence of the *ced-4* mutation did not reduce the statin resistance in the *nduf-7(et19)* mutant. *** indicates $p < 0.001$ in a Student's t-test.
